# On Bayesian modeling of censored data in JAGS

**DOI:** 10.1186/s12859-021-04496-8

**Published:** 2022-03-23

**Authors:** Xinyue Qi, Shouhao Zhou, Martyn Plummer

**Affiliations:** 1 The University of Texas MD Anderson Cancer Center, Houston, TX USA; 2 Pennsylvania State University, Hershey, PA USA; 3 University of Warwick, Coventry, UK

**Keywords:** Bayesian data analysis, Survival analysis, Deviance function, Deviance information criterion, Exact likelihood, Model selection

## Abstract

**Background:**

Just Another Gibbs Sampling (JAGS) is a convenient tool to draw posterior samples using Markov Chain Monte Carlo for Bayesian modeling. However, the built-in function dinterval() for censored data misspecifies the default computation of deviance function, which limits likelihood-based Bayesian model comparison.

**Results:**

To establish an automatic approach to specifying the correct deviance function in JAGS, we propose a simple and generic alternative modeling strategy for the analysis of censored outcomes. The two illustrative examples demonstrate that the alternative strategy not only properly draws posterior samples in JAGS, but also automatically delivers the correct deviance for model assessment. In the survival data application, our proposed method provides the correct value of mean deviance based on the exact likelihood function. In the drug safety data application, the deviance information criterion and penalized expected deviance for seven Bayesian models of censored data are simultaneously computed by our proposed approach and compared to examine the model performance.

**Conclusions:**

We propose an effective strategy to model censored data in the Bayesian modeling framework in JAGS with the correct deviance specification, which can simplify the calculation of popular Kullback–Leibler based measures for model selection. The proposed approach applies to a broad spectrum of censored data types, such as survival data, and facilitates different censored Bayesian model structures.

## Introduction

Censored data are commonly observed in different disciplines such as economics, engineering and life sciences [1–3]. Given the uncertainty in censored data, the modeling and analysis fit naturally in the Bayesian framework by using expectation-maximization (EM), data-augmentation (DA) and Markov chain Monte Carlo (MCMC) algorithms [4, 5]. For example, in highly fractionated experiments, frequentist likelihood-based estimates may not even exist for simple models consisting of only main effects, while Bayesian approach offers a straightforward implementation strategy [6]. When the outcome cannot be fully observed, censored data can be treated as additional parameters from a fully Bayesian perspective, with a likelihood function specifying joint modeling for both observed and censored data. The Bayesian approach has multiple advantages in the presence of censored data or inadequate sample size, and for nested/non-nested model comparisons [7]. Compared with the multiple imputation, Bayesian modeling is robust in statistical inference even when a large proportion of missing data is present [8, 9].

Just Another Gibbs Sampling (JAGS) is an object-oriented software to generate posterior samples using MCMC simulations [10]. It avoids the explicit specification of the MCMC algorithms for model parameters, especially when the closed-form expressions of conditional distributions are not available, and simplifies the implementation of Bayesian modeling. JAGS also clarifies certain confusing aspects for missing data in BUGS [11, 12]. To distinguish the concepts of censoring and truncation, it introduces a degenerate dinterval distribution function for general interval-censored data [10].

Some existing R packages, including rjags [13], r2jags [14] and runjags [15], provide a user-friendly interface for R users to conduct Bayesian data analysis via JAGS. Most importantly, these R packages for JAGS, together with coda [16] and MCMCpack [17], not only make it easy to process the output of Bayesian models implemented using JAGS, but also further help (1) visualize the posterior samples via plots, (2) predict new data based on posterior predictive distributions, and (3) calculate the deviance using posterior samples from JAGS models.

For Bayesian inference especially with complicated model features, model selection is a critical component to identify an approximate model best describing the information in the data. Among many popular approaches, the seminal work of deviance information criterion (DIC) by [18] was proposed based on Kullback–Leibler (K–L) divergence [19] and embedded in JAGS as part of the dic module based on the posterior samples obtained from MCMC simulations. However, when the outcome variables are censored, the built-in function dinterval() returns a constant value of 1 for the likelihood calculation [20, 21], which is equivalent to ignoring all of the censored observations in the deviance monitor of the dic module. As a result, it fails to calculate DIC for model comparison, which may limit the broader usage of JAGS for Bayesian modeling of censored data [22].

Therefore, we propose an alternative model specification for the analysis of censored outcomes in JAGS. It is a universal approach that automatically returns the correct deviances for both observed and censored data, such that DIC and penalized expected deviance [23] can be properly and simultaneously calculated using posterior samples from MCMC simulations; thus Bayesian model selection for censored data modeling can be conducted using JAGS without analytical customization of the deviance of the model. The proposed approach is applicable to many different Bayesian model structures, such as Bayesian tobit regression model [24], semiparametric accelerated failure time (AFT) models for censored survival data [25], illness-death model using Bayesian approach for semicompeting risks data [26], Bayesian hierarchical model for censored normal outcome [27], and Bayesian Thurstonian models for ranking data [28], among many.

The rest of the paper is organized as follows. We first introduce the default approach for censored data modeling using the built-in function in JAGS, and then we propose an alternative strategy for correct deviance computation. Furthermore, we use a right-censored survival example to illustrate the discrepancy in deviance functions using both approaches, and apply Bayesian model selection using the correctly specified likelihood in an application to drug safety for cancer immunotherapy. Concluding remarks and discussions are given at the end.

## Default procedure for censored data modeling in JAGS

Censoring occurs when the value of an observation is only partially observed, which is common in Bayesian modeling. Hereinafter we assume that the outcome model and the censoring mechanism are independent, a.k.a. noninformative censoring in survival analysis. It is a fundamental assumption for censored data behind most statistical methodologies [29]. We first briefly review the standard approach to model censored data in JAGS with its limitation in model assessment.

A default approach for analysis of censored observations in JAGS is to use the built-in dinterval distribution function for model specification and posterior sampling. The *Model 1* below illustrates a general form of model specification for censored data analysis in JAGS. It helps to model three types of censoring: right-censoring, left-censoring and interval-censoring [21].

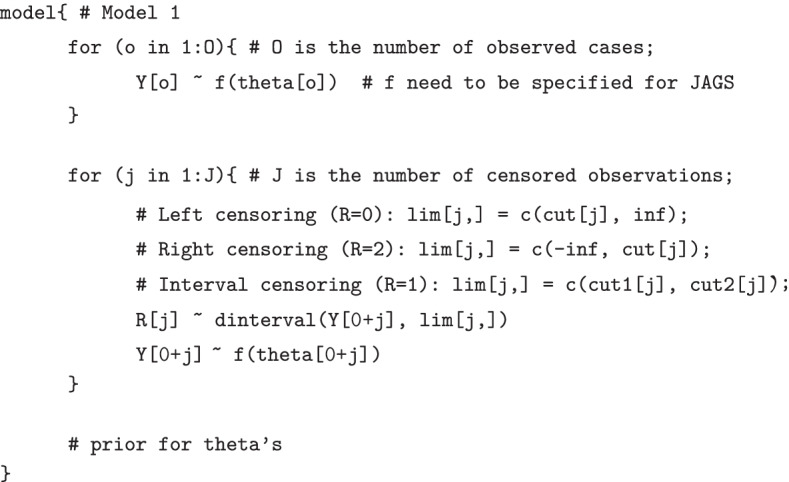



where the outcome of interest, *Y*, which can be either observed or censored (coded as $\texttt {NA}$ in the data table), follows density distribution f with parameter $\theta$. *R* is a censoring variable following an interval distribution. If $R=1$, then the outcome is interval-censored; cut1[] and cut2[] are lower and upper cutoff values for interval-censoring, respectively. If $R=0$, the data is left-censored while the outcome contains partial information which is less than a lower limit; If $R=2$, the data is right-censored, which is above a certain cutoff value. lim[,] is a vector of length 2, which contains a pair of cutoff values for each unobserved outcome data, as illustrated in the comment lines above, and cut[] specifies the one-sided cutoff value for left/right-censoring.

However, dinterval() function has a limitation in deviance calculation when we assess model fit based upon deviance-based statistics. For example, when we apply an existing function, dic.samples(), in the rjags package [13] to call the dic module and to generate penalized deviance samples within R [30], the following warning message appears.

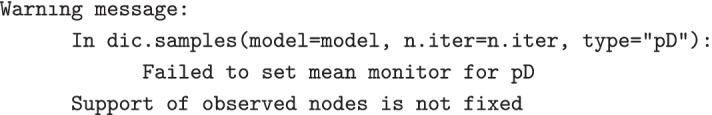



By default, the dic module was created to monitor and record the likelihood/deviance of a JAGS model at each iteration and calculate the deviance-based model selection criteria such as DIC or penalized expected deviance. In the presence of censored outcomes, even though the dinterval() function can generate the proper posterior distribution of the parameters in JAGS, the likelihood function is misspecified with *the wrong focus* of inference on the censored outcome variable [22]. Instead, a constant value of 1 for the likelihood function, or equivalently, a constant value of 0 for the deviance function, is misspecified for the censored outcomes in the deviance monitor. Therefore, the posterior mean deviance computed from the dic module using the default procedure dinterval() is mistakenly reported by the posterior mean deviance of observed data only; see also the first example in **Illustrative Examples**. It suggests that the posterior mean deviance extracted from the dic module in JAGS should not be used in model assessment [20].

## Alternative modeling strategy in JAGS

The goal is simply to derive the deviance and associated model selection criteria in JAGS without any manual calculation by definition. Rather than handling censored data with the dinterval() function in the JAGS *Model 1*, we present an alternative modeling strategy to specify the proper deviance based on the type of censoring.

We divide the data into 3 subgroups: observed, left- or right-censored, and interval-censored. For incomplete observations, we introduce ancillary indicator variables $Z_1$ for left- and right- censored data and $Z_2$ for interval-censored data. Hence, the alternative JAGS model specification (*Model 2*) can be written in a general form as follows:
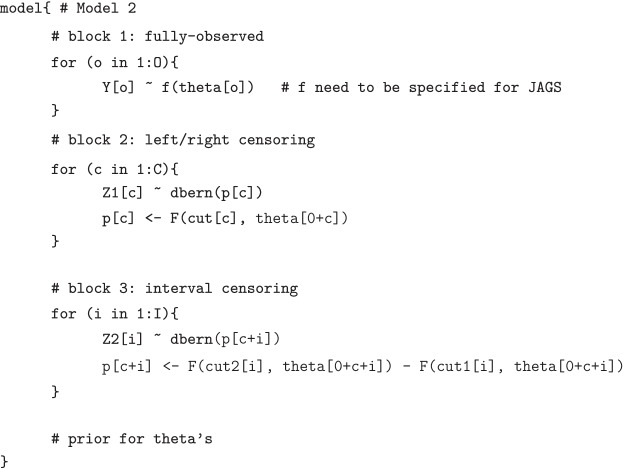


Every subgroup is self-blocked with a separate section of the likelihood in JAGS, where *O* is the set of observed data, *C* is the set of left/right-censored observations, and *I* is the set of interval-censored observations. $Z_1$ is a binary random variable, where $Z_1=1$ if it is left-censored, or $Z_1=0$ if right-censored. The probability of success *p* in the Bernoulli distribution of $Z_1$ is defined by the cumulative distribution *F* for the censored outcomes, which neatly identifies the probabilities for both left-censored and right-censored data with properly specified cutoffs. For interval censored observations, we set $Z_2=1$ and the probability of success in Bernoulli distribution is the incremental change of the values in *F* function between the cutoffs, corresponding to the unobserved outcome which lies in a semi-closed interval.

Proposition 1 in “Appendix A” demonstrates that the proposed alternative modeling strategy in the JAGS *Model 2* has a correctly specified likelihood function for censored data. Therefore, it is warranted that the JAGS *Model 2* can generate proper posterior samples and deliver valid Bayesian posterior inference.

In addition, the JAGS *Model 2* spontaneously specifies correct deviances in the dic module for model assessment of censored observations. For K-L based model comparison, especially when there are complicated model features, it is convenient to have an automatic algorithm to avoid any manual calculation of deviance function and model selection criteria. Because the computation is implemented *via* the built-in dic module, we empirically compare the deviance reported from the JAGS *Model 2* to the deviance manually calculated using posterior samples in the next section and illustrate that the proposed model can report the correct deviance values.

The JAGS *Model 2* encompasses a broad range of model structures. The censored regression models, which are also called tobit models, usually have data both in blocks 1 and 2 with normally distributed or *t*-distributed errors [24, 31]. Some extensions include time-series analysis [32], longitudinal data analysis [33] and spatial analysis [34]. In the context of survival data analysis, some commonly assumed parametric distributions *F* include exponential, Weibull, generalized gamma, log-normal, and log-logistic [35, 36], since the event times are positively valued with a skewed distribution, making the symmetric normal distribution a poor choice for fitting the data closely. Additionally, it is unnecessary to assume a known censoring time. Because the cutoff can be either pre-specified with a fixed value or modeled as a random variable, the proposed approach naturally accommodates models with unobserved, stochastic censoring thresholds [37].

The proposed modeling strategy coincides with non-censored discrete data modeling in some situations for computational advantages. After converting the standard model to a latent-variable formulation, we can adapt logit, probit or complementary log-log models as a type of block 2 data with $Z_1$ defined as the binary outcome and cut (cutoff) treated as fixed at 0 [38]. It is also possible to extend the proposed approach for ordered probit analysis [39], which accommodates many applications in economics and marketing [40].

## Illustrative examples

In this section, two real data applications are examined with the proposed approach. The first example applies both the default approach and the alternative strategy to model time-to-event outcomes with right censoring. The reported deviance of the model is assessed with the true value calculated manually based on the full likelihood function. It demonstrates that the alternative strategy not only properly draws posterior samples in JAGS, but also automatically delivers the correct deviance for model assessment. The second example shows that the proposed approach is capable of comparing censored data models by DIC [18] and penalized expected deviance (PED, [23]) simultaneously, using a drug safety subset [41] in which some of the outcome data are left-censored.

### Survival data

Right censoring is common in the time-to-event data of survival analysis. The first example is from a classical right-censored survival dataset on acute myeloid leukemia [42]. Individual patient-level data were collected along with survival or censoring time to test whether the standard course of chemotherapy should be maintained for additional cycles or not. The Bayesian survival analysis is conducted using MCMC simulation and implemented in JAGS 4.3.0 software [21] and R version 3.4.1. The JAGS codes for both models are attached in “Appendix B”. We run three parallel chains for the exponential survival regression model and discard the first 30,000 iterations of burn-in, followed by saving 10,000 posterior samples of parameters per MCMC chain with thinning by 3. Once the posterior samples are obtained, the deviance function of the model based on the exact likelihood function is manually calculated, and compared with the calculated deviance using dic.samples() function in the rjags package with additional 10,000 iterations.

**Fig. 1:**
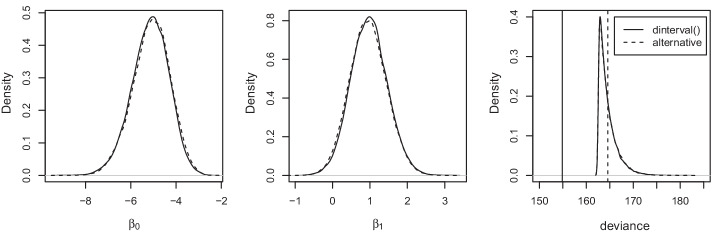
**a** A kernel density plot of regression coefficient $\beta _0$ (the log of the baseline hazard) in the exponential survival regression model comparing two methods; **b** a kernel density plot of regression coefficient $\beta _1$ (the log of the hazard ratio in patients who maintain additional cycles of chemo relative to patients who do not) comparing two methods; **c** a kernel density plot of deviance functions comparing two methods by manual computation of deviance from posterior samples (based upon the exact likelihood). The two vertical lines show the mean deviances generated via the dic.samples() function by the two methods

The deviance information criterion (DIC; [18]) for model comparison is the posterior mean deviance plus the effective number of parameters as below,

$$DIC = \overline{D(\lambda)} + p_{D}$$

where the deviance function *D*(*λ*) for this example is given by

$$D(\lambda) = D_{obs}(\lambda)+D_{cen}(\lambda)= -2\left[ {\sum\limits_{{o = 1}}^{O} {\log f_Y(y_{o} |\lambda ) + \sum\limits_{{c = 1}}^{C} {\log (1 - F_{Y} (y_{c}^{ - } |\lambda ))} } } \right]$$

where *O* = 18 for the observed cases and *C* = 5 for the censored cases. By definition, we manually calculate the DIC values for the exponential survival regression model using the posterior samples with $\overline{{D(\lambda )}} = 164.6$, which is exactly the same as the posterior mean deviance obtained from dic module using our proposed approach. In contrast, the default approach using dinterval() leads to a mean deviance of 154.9, which is in fact the mean deviance of observed data only ($\overline{{D_{obs}(\lambda )}} = 154.9$), suggesting that the dic module is prone to a *wrong focus* on the censored outcome and mis-specifies the deviance function. This demonstration entails the key distinction between the proposed and default approaches on the *correct/wrong* focus of dic module to consider both observed and censored data.

Figure 1a on the left and Fig. 1b in the middle compare the kernel density plots of posterior samples for coefficients in the exponential survival regression model between the default approach using dinterval() and the alternative strategy. The proposed approach has almost identical distributions to the default approach using dinterval() in estimation of the coefficient parameters. The output of dic.samples() function for mean deviance estimation is plotted in Fig. 1c on the right, where the solid vertical line shows the mean deviance using dinterval() function and the dashed vertical line using the proposed alternative strategy. Based on the saved MCMC samples, we also manually calculate the deviance based on the exact likelihood (1) and plot their kernel density curves displayed in the last panel. The result demonstrates that the proposed JAGS *Model 2* provides the correct value of mean deviance, while the estimate using dinterval() function is significantly biased due to the deviances ignored for censored outcomes.

### Binomial data

The second example is from an application to assess drug safety for cancer immunotherapy, known as programmed cell death protein 1 (PD-1) and programmed death-ligand 1 (PD-L1) inhibitors. In clinical practice, it is important to investigate the incidence of treatment-related adverse events (AEs) and to better understand the safety profiles of these immuno-oncology drugs. In this illustrative example, we apply the alternative strategy after extracting all-grade pneumonitis (a specific type of AE for inflammation of lung tissue) data from a recent meta-analysis [41]. The primary response is a binomial outcome for the number of pneumonitis cases that could be censored; some rare pneumonitis data may be missing due to low incidence. Usually, the less frequently observed AEs are less likely to be disclosed, given the prevalent manuscript word count limitations for clinical trial publications in medical journals. For each censored AE, a study-specific cutoff value can be identified; only the AEs either of special interest or with observed incidence exceeding the cutoff were reported. To take those non-ignorable censored data into account, we considered study-level rare binomial AE outcome data within the data coarsening framework [43] to examine the impact of stochastic censoring mechanism. If the data are coarsened at random, then we can construct the resultant likelihood ignoring the coarsening mechanism and model the outcome data only, as is presented below. The complete likelihood can be represented and modeled using *selection model* factorization including sensitivity analysis [44]. More technical details can be found in [45].

In the Bayesian context, we compare seven distinct censored binomial models for all-grade pneumonitis data to examine the model performance using the proposed strategy. To apply the JAGS *Model 2*, an outcome variable $Z_1$ is incorporated for censoring status in block 2. In Model A, a baseline beta-binomial model by complete pooling is to estimate the overall incidence of AE, in which no additional effect is included. In Model B, two-group drug effect is incorporated into the baseline model, and then we can estimate the AE incidences for two drug groups (PD-1 vs. PD-L1 inhibitors). To allow for five drug-specific (Nivolumab vs. Pembrolizumab vs. Atezolizumab vs. Avelumab vs. Durvalumab) effect on the incidence of AE, we begin with modeling drug effects without any link function as Model C, and then extend to specify half-Cauchy prior [46] to the standard deviation of drug effect with logit, cloglog, and probit link functions in Model D-F, respectively. Lastly, we include a saturated model G to estimate the incidence rate corresponding to each study without pooling. Mean deviance (${\bar{D}}$), effective number of parameters ($p_D$), DIC, optimism ($p_{opt}$), and PED are all calculated and compared based on the seven candidate models described above. The model assessment results obtained from the proposed JAGS models are summarized in Table 1.

**Table 1:** Model comparison: posterior mean deviance (${\bar{D}}$), effective number of parameters ($p_D$), deviance information criterion (DIC), optimism ($p_{opt}$), and penalized expected deviance (PED) from modeling observed and censored all-grade AE (pneumonitis) data

Model	${\bar{D}}$	$p_D$	DIC	$p_{opt}$	PED
A	380.85	0.99	381.84	2.05	382.90
B	371.11	1.99	373.10	4.26	375.37
C	343.14	4.61	347.75	10.65	353.79
D	343.35	4.56	347.91	11.02	354.37
E	343.39	4.54	347.93	13.19	356.58
F	343.38	4.61	347.99	10.28	353.66
G	269.30	94.60	363.90	865.69	1134.99

Per the results summarized in Table 1, there is no significant discrepancy on either DICs or PEDs between Model C–F, indicating that the data are not sensitive to the choice of link functions. In general, models with drug-specific effects (Model C–F) outperform the baseline model with complete pooling (Model A) and the model with PD-1/PD-L1 effect (Model B); the beta-binomial model without pooling (Model G) overfits the data. All results are simultaneously computed from dic.samples() function in the rjags package from R.

## Discussion

In this paper we propose an alternative strategy to apply Bayesian modeling for censored data in JAGS. It specifies the correct deviances for censored observations such that the model selection methods DIC and PED can be easily calculated from the built-in dic module. This approach can also simplify the calculation of other popular Bayesian K-L based measures such as the Bayesian predictive information criterion (BPIC, [47]) and the widely applicable information criterion (WAIC, [48]). Though not explicitly specified, the proposed approach can be easily extended to model truncated data, for example, left-truncated right-censored observations in survival analysis. Even for non-censored data such as binary outcomes, the proposed approach can still be useful for computational advantages.

The proposed method may have a similar model presentation to the EM algorithm [4] to handle censored data, for example, in tobit or probit regression modeling [49, 50]. In Bayesian contexts, the EM-type algorithms are designed to apply parameter optimization in the posterior mode estimation, while the goal is to achieve the automatic calculation of deviance with the posterior distribution estimation. DA is another relevant approach to estimate the posterior distribution, which constructs computationally convenient iterative sampling via the introduction of unobserved data or latent variables [5, 24, 39]. Different from our approach, DA requires the sampling of the unobserved data, which may alter the deviance in application of K-L based model selection [18].

A relevant question, as raised by a reviewer, is how the miscalculated DIC value may impact model comparison. In a data analysis project, a ranking of candidate models can be derived based on the comparison of the calculated DIC values from dic module in JAGS. If the DIC is used for model selection, can the default method and the proposed approach make any difference in the ranking, or equivalently, the selected model? From a modeling perspective, there are scenarios to yield the exact identical DIC ranking using both default and alternative JAGS model specifications, only if the additional deviance of the censored data doesn’t change the ranking using deviance of observed data only. However, the major contribution of this work is not to distinguish in which scenarios there could be a discrepancy, but to propose a care-free approach that can always deliver the correct model ranking to facilitate the appropriate model selection.

Censoring is frequently observed in real-world data analysis. In addition to normally distributed data in censored regression models, various types of outcome, including survival data [7], binomial data [41], count data [51] and ranking data [28], can all be modeled by the proposed alternative strategy when censoring occurs. Not only to the medical sciences, the proposed strategy can also be applied to many other fields, such as, in measuring the performance of timing asynchronies using censored normal sensorimotor synchronization data in behavioral science [52], comparing industrial starch grain properties with ordered categorized data in agriculture [53], exploring forest genetics by modeling censored growth strain data for narrow-sense heritability estimation in environmental science [54], determining the importance of influential factors to lower the risk of food contamination for censored microbiological contamination data in food science [55], modeling the interval-censored as well as right-censored time to dental health event in primary school children for public health science [56], and modeling the demand data related to the supply chain management when the distribution of demand could be censored by inventory [57]. In summary, the proposed JAGS model specification can encompass a broad range of popular model structures and be utilized in a wide spectrum of applications.

## Data Availability

The survival data used in the first illustrative example are openly available in the R package “survival” v3.2-11. The binomial data and model that support the findings of the second illustrative example are openly available at https://github.com/xinyue-qi/Censored-Data-in-JAGS.

## Appendix A: Alternative modeling strategy

We justify that the proposed alternative procedure constructs the correct likelihood function for censored outcomes. In likelihood-based inference, the full likelihood for observed and censored data comprises four key components: observed case, left-censored case, right-censored case and interval-censored case. For observed data, the likelihood is simply a product of individual probability density/mass function of the observed outcome. For any type of censored cases, the likelihood can be presented in a form of $F_{Y}(b)-F_{Y}(a)$, defining the probability of a censored outcome *Y* observed in the semi-closed interval, (*a*, *b*]. Here, $F_Y(y)=\text {P}(Y\le y)$ denotes the cumulative distribution function of the random outcome variable if it is fully observed. If the outcome variable is left-censored at a cutoff, $y_l$, then $F_{Y}(b) = F_{Y}(y_l)$ and $F_{Y}(a) = F_{Y}(-\infty ) = 0$. If data is right-censored with a lower bound, $y_r$, then $F_{Y}(a) = F_{Y}(y^-_r)$ and $F_{Y}(b) = F_{Y}(+\infty )= 1$. For interval-censored data, the likelihood function is the product of $\text {Pr}(u_i \le Y \le v_i) = F_Y(v_i) - F_Y(u^-_i)$, where $u_i$ and $v_i$ are a pair of interval thresholds, which could vary for every observation. Therefore, the exact likelihood function is given by:

1 $$\begin{aligned} {\mathcal {L}}_{exact}\left( \theta ; y \right) = \prod _{o \in O}f_Y\left( y_o \right) \prod _{l \in L}F_Y\left( y_l \right) \prod _{r \in R} \left[ 1-F_Y\left( y^-_r \right) \right] \prod _{i \in I} \left[ F_Y\left( v_i \right) -F_Y\left( u^-_i \right) \right] , \end{aligned}$$

where *O* is the set of observed outcome, *L* (or *R*) is the set of left (or right) censored observations, and *I* is the set of interval-censored data with $u_i$ and $v_i$ denoting the lower and upper bound of the *i*th interval-censored observation.

In the JAGS *Model 2*, we can specify the cutoff value cut = $y_l$ if data are left-censored, cut = $y^-_r$ if data are right-censored, and (cut1, cut2) = $(u^-_i, v_i)$ if data are interval censored. Defining F = $F_Y$, we have the following property for the likelihood from the proposed JAGS model.

### Proposition 1

*The likelihood generated from the JAGS* *Model 2* *using Bernoulli distribution with the cumulative probabilities for censored data is identical to the exact likelihood* (1).

### Proof

To illustrate that the likelihood from the JAGS *Model 2*, ${\mathcal {L}}_{jags}$, is identical to its exact likelihood, ${\mathcal {L}}_{exact}$, we start with deriving the formula for the likelihood presented in the censored JAGS model, which has three major components: observed case, one-sided censored case, and interval-censored case. The full likelihood, ${\mathcal {L}}_{jags}$, can be written as:

2 $$\begin{aligned} \begin{aligned} {\mathcal {L}}_{jags}\left( \theta ; y \right)&= \prod _{o \in O}f_Y\left( y_o \right) \prod _{c \in C} \left\{ \left[ F\left( \texttt {cut}_{c} \right) \right] ^{I\left( Z_{1,c}=1 \right) } \left[ 1-F\left( \texttt {cut}_c \right) \right] ^{I\left( Z_{1,c}=0 \right) } \right\} \\&\quad \prod _{i \in I} \left[ F\left( \texttt {cut2}_i \right) -F\left( \texttt {cut1}_i \right) \right] ^{I\left( Z_{2,i}=1 \right) } \\&= \prod _{o \in O}f_Y\left( y_o \right) \prod _{\begin{array}{c} c \in C \\ \left\{ Z_{1,c}=1 \right\} \end{array}} F\left( \texttt {cut}_{c} \right) \prod _{\begin{array}{c} c \in C \\ \left\{ Z_{1,c}=0 \right\} \end{array}} \left[ 1-F\left( \text {cut}_{c} \right) \right] \\&\prod _{\begin{array}{c} i \in I \\ \left\{ Z_{2,i}=1 \right\} \end{array}} \left[ F\left( \texttt {cut2}_{i} \right) -F\left( \texttt {cut1}_{i} \right) \right] \\&= \prod _{o \in O} f_Y\left( y_o \right) \prod _{l \in L} F_Y\left( y_l \right) \prod _{r \in R} \left[ 1-F_Y\left( y^-_r \right) \right] \prod _{i \in I} \left[ F_Y\left( v_i \right) -F_Y\left( u^-_i \right) \right] . \end{aligned} \end{aligned}$$

$$\square$$

## Appendix B: JAGS code for survival example

The following is the JAGS code for survival regression model.
