# Correction: On Bayesian modeling of censored data in JAGS

**DOI:** 10.1186/s12859-022-04785-w

**Published:** 2022-06-17

**Authors:** Xinyue Qi, Shouhao Zhou, Martyn Plummer

**Affiliations:** 1 The University of Texas MD Anderson Cancer Center, Houston, TX USA; 2 Pennsylvania State University, Hershey, PA USA; 3 University of Warwick, Coventry, UK

## Correction to: BMC Bioinformatics (2022) 23:102 https://doi.org/10.1186/s12859-021-04496-8

Following the publication of the original article [1], the authors identified errors in the model specifications 1 and 2. The correct models are given below.
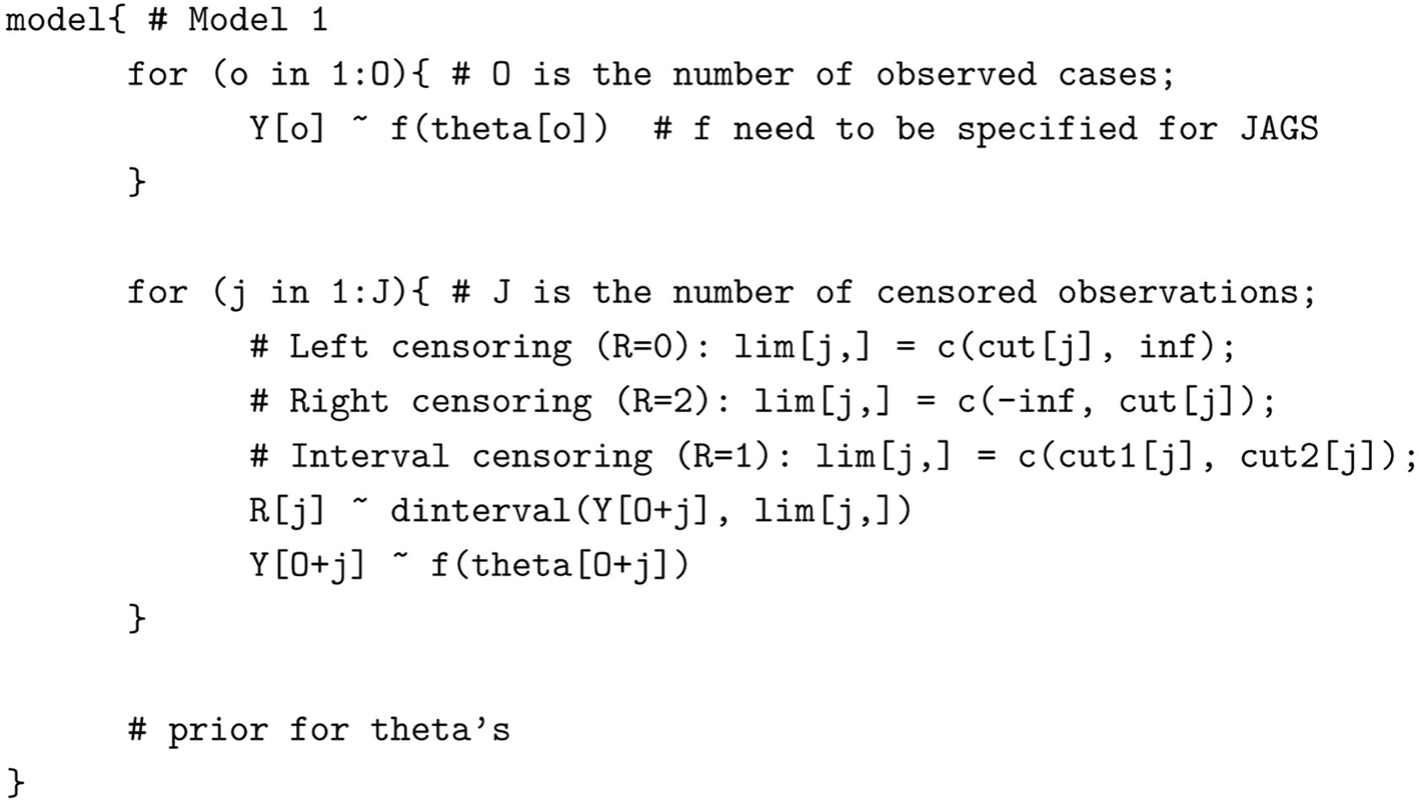

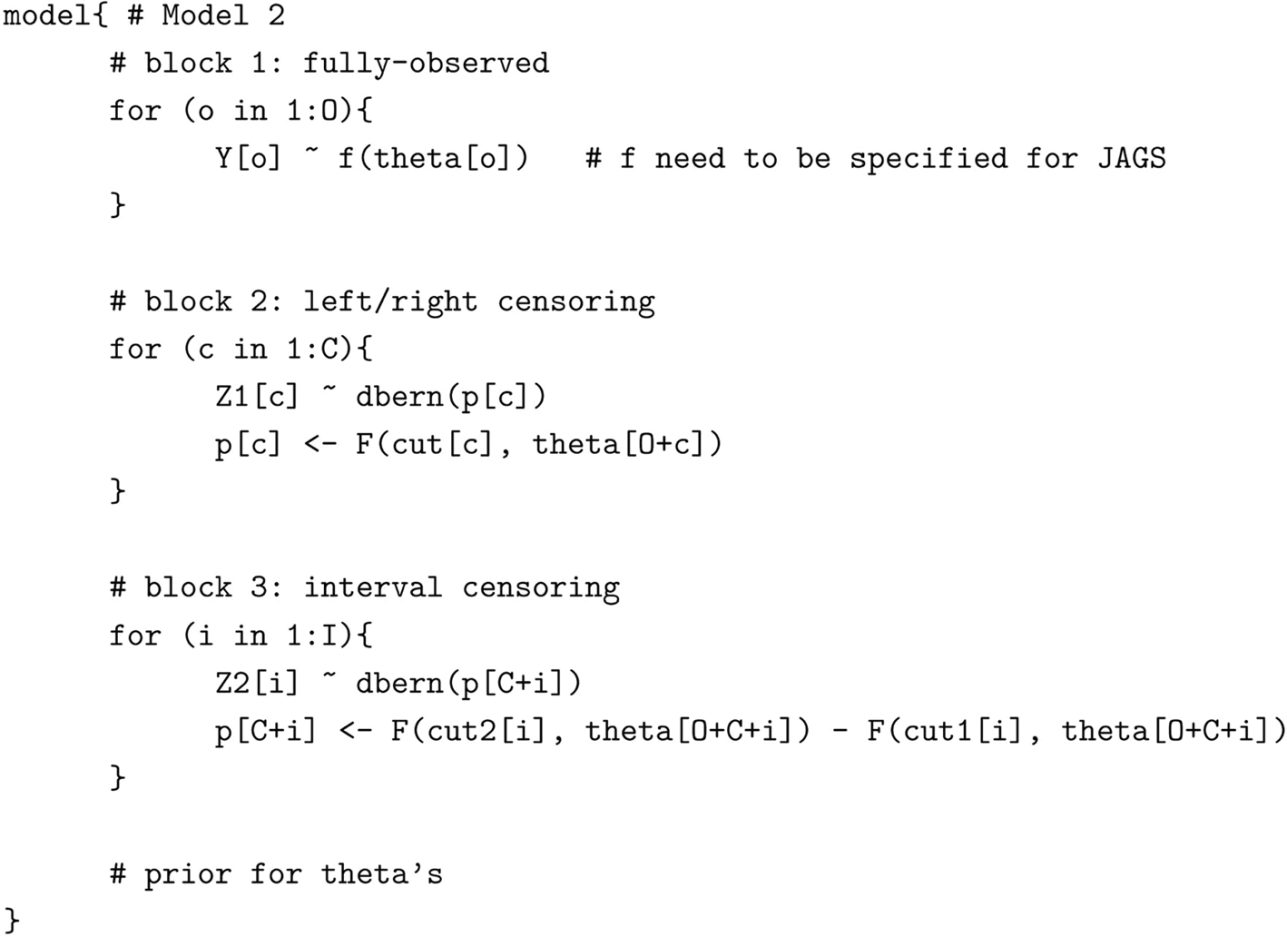


The original article [1] has been corrected.
